# Protein-mediated Fatty Acid Uptake in the Heart

**DOI:** 10.2174/157340308783565429

**Published:** 2008-02

**Authors:** Adrian Chabowski, Jan Górski, Jan F.C Glatz, Joost J. F P Luiken, Arend Bonen

**Affiliations:** 1 Department of Physiology, Medical University of Bialystok, 15-089 Bialystok, Poland; 2 Department of Molecular Genetics, Maastricht University, 6200-MD Maastricht, The Netherlands; 3 Department of Human Health and Nutritional Sciences, University of Guelph, Guelph, Ontario, N1G 2W1, Canada

**Keywords:** Fatty acid transport, FAT/CD36, FABPpm, heart.

## Abstract

Long chain fatty acids (LCFAs) provide 70-80% of the energy for cardiac contractile activity. LCFAs are also essential for many other cellular functions, such as transcriptional regulation of proteins involved in lipid metabolism, modulation of intracellular signalling pathways, and as substrates for membrane constituents. When LCFA uptake exceeds the capacity for their cardiac utilization, the intracellular lipids accumulate and are thought to contribute to contractile dysfunction, arrhythmias, cardiac myocyte apoptosis and congestive heart failure. Moreover, increased cardiac myocyte triacylglycerol, diacylglycerol and ceramide depots are cardinal features associated with obesity and type 2 diabetes. In recent years considerable evidence has accumulated to suggest that, the rate of entry of long chain fatty acids (LCFAs) into the cardiac myocyte is a key factor contributing to a) regulating cardiac LCFA metabolism and b) lipotoxicity in the obese and diabetic heart. In the present review we i) examine the evidence indicating that LCFA transport into the heart involves a protein-mediated mechanism, ii) discuss the proteins involved in this process, including FAT/CD36, FABPpm and FATP1, iii) discuss the mechanisms involved in regulating LCFA transport by some of these proteins (including signaling pathways), as well as iv) the possible interactions of these proteins in regulating LCFA transport into the heart. In addition, v) we discuss how LCFA transport and transporters are altered in the obese/diabetic heart.

## INTRODUCTION

Long chain fatty acids (LCFAs) provide 70-80% of the energy for cardiac contractile activity [1,2]. LCFAs are also essential for other processes, such as genetic reprogramming *via* activation of transcription factors regulating the transcription of enzymes involved in lipid metabolism [3]. They serve as substrates for membrane synthesis and can modulate many intracellular signaling pathways [1,2]. Inside cardiac myocytes there is a limited capacity for lipid storage, and hence, the uptake and oxidation of LCFA are tightly coupled [2]. Although LCFA can enter the cells *via* simple diffusion [4-8], considerable evidence has accumulated in recent years showing that a protein-mediated mechanism is involved in the uptake of myocardial LCFA [for recent reviews see: 4, 9-11]. Hence, there is a considerable interest in elucidating the regulation of LCFA movement into the myocardium *via* protein-mediated LCFA transporters. For both LCFA uptake and oxidation, specific LCFA binding proteins play a substantial role in facilitating LCFA movement across cellular membranes (plasma membranes and mitochondrial membranes, respectively). Although the precise mode of action of these proteins is not yet known, for convenience they are referred to as LCFA transport proteins or LCFA transporters [4]. By regulating the rate of the entry of LCFAs into the cardiac myocytes, these LCFA transport proteins may serve as a key factor contributing to the regulation of LCFA metabolism in healthy hearts, while in obesity and diabetes these proteins may contribute to the lipotoxicity associated with excess lipid accumulation in the heart.

To date, it has been shown that myocardial expression of LCFA transporters is modulated through both transcriptional and post-transcriptional mechanisms [see review: 11]. Another level of regulation is the distribution of these transporters between intracellular compartments and the plasma membrane, which determines the myocardial capacity for LCFA uptake. Several LCFA transporters are expressed in the heart, including fatty acid translocase (FAT)/CD36, plasma membrane associated fatty acid binding protein (FABPpm) and fatty acid transport proteins 1 and 6 (FATP1 and 6) [12-16]. Solid evidence confirming the roles of these various proteins as fatty acid transporters are based on genetic studies. In mice, ablation of myocardial FAT/CD36 reduced the rate of LCFA uptake and lowered the basal rates of LCFA esterification and oxidation in heart [17-19]. However, compensatory substrate flexibility was observed, because FAT/CD36-null hearts switched to glucose oxidation to sustain normal cardiac energetics [19]. Overexpression of FABPpm in skeletal muscle, *via* electrotransfection of the corresponding cDNA into soleus muscle, increased the rate of LCFA transport into this muscle [20]. In cardiospecific FATP-1 overexpressor mice, there were increased rates of palmitate esterification and oxidation, along with a decreased rate of glucose utilization [21], while in FATP1 KO mice, insulin-stimulated triacylglycerol synthesis was blunted [22], and diet-induced insulin resistance was prevented [23]. Importantly, genetic approaches in Saccharomyces cerevisiae have shown that the transport capacities of the FATP isoforms differ markedly [24]. For example, the LCFA transport capacity of FATP-4 was greater than that of FATP-1, and the transport capacities of both FATP-1 and 4 greatly exceeded that of FATP-6 [24]. Thus, the LCFA transport role of FATP-6 in the heart may be less important than initial studies [25] had suggested. In general little is known about the physiologic regulation of FATPs in the heart, while considerably more is known about the regulation of FAT/CD36 in this organ.

## MYOCARDIAL UPTAKE AND METABOLISM OF LCFA

LCFA uptake into cardiac myocytes and their subsequent metabolism has been recently reviewed [9,10,26] and only a brief summary is presented here. As mentioned above, myocardial LCFA uptake occurs by two different processes. First, based on the hydrophobic nature of LCFAs, they may traverse the lipid bilayer of the cardiac myocytes’ sarcolemma by simple diffusion [4-7]. However, it has been shown that myocardial, protein-mediated LCFA transmembrane transport accounts for more than 70% of total cardiac LCFA uptake [17,19,27,28].

Little is currently known regarding the exact mechanism by which protein-mediated LCFA trafficking occurs across the plasma membrane. Possibly, these proteins function in trapping blood-borne LCFAs and transmembrane translocation of LCFA occurs by a flip-flop mechanism [4,9,10]. Kleinfeld’s group has attempted to address this matter recently [8], but full details of the transmembrane LCFA movement process remain unknown. Presently, it is also unclear why there are a number of different LCFA transporters. They are derived from separate genes and are structurally unrelated [29-31]. It is not known whether there is one LCFA transporting system involving the concerted action of all the fatty acid transporters, or alternatively, whether each of these proteins is separately involved in transmembrane LCFA transport, possibly targeting LCFAs to specific metabolic processes within the cell (Fig. (**1**)).

Once LCFAs have crossed the sarcolemma, they are transferred to cytoplasmic heart-type fatty acid-binding protein (H-FABPc). FABPs are small (15 kDa), highly conserved cytoplasmic proteins that bind long-chain fatty acids with 1 : 1 molar stoichiometry and micromolar affinity [32,33]. This binding of LCFAs to the intracellular H-FABPc likely occurs through protein-protein interaction between FAT/CD36 and H-FABPc, as both proteins coimmunoprecipitate in cellular lysates, at least in epithelial mammary gland cells [34]. The intracellular H-FABPc capacity for binding of LCFAs controls movement and concentration of this lipid fraction. Thus, binding with H-FABPc is an important player in cellular LCFA uptake. Data from FABPc KO mice support involvement of FABPc in shuttling the FA moieties from sarcolemma to the target sites of oxidation or esterification [35-38]. Under normal circumstances FABPc is well in excess of the LCFAs transported into muscle cells [39].

Mammals express multiple isoforms of acyl-CoA synthetase (ACSL1 and ACSL3-6), and they are expressed differentially in various tissues [40]. It has been postulated that these enzymes are essential for LCFA metabolism, providing activated intermediates for complex lipid synthesis and β-oxidation [41]. Interestingly, one of the LCFA transporters, namely FATP1, displays acyl-CoA synthetase activity [42,43], but this activity does not seem to account for the transport activity of FATP1 or other FATPs [24]. Schaffer has speculated [44,45] that there may be fatty acyl-CoA synthetases associated with FAT/CD36 on the cytosolic side of the sarcolemmal membrane. This direct (FATPs) or indirect (FAT/CD36) linkage of LCFA transporters and fatty acyl-CoAs, indicates that a considerable portion of LCFAs transported across the membrane are rapidly esterified into fatty acyl-CoA. The latter process not only prevents backflux of LCFAs but also commits the esterified LCFAs (fatty acyl-CoA) to further metabolism. These long-chain acyl-CoAs are immediately complexed with acyl-CoA binding proteins (ACBP) [46], which possess high affinity for long-chain fatty acyl-CoAs. ACBPs are indispensable for efficient transcytoplasmic trafficking of long-chain fatty acyl-CoAs [35-37,46]. The fate of intracellular fatty acyl-CoAs is largely determined by the rates of their transport into mitochondria, β-oxidation, and Krebs cycle flux. In the heart cytosol, the complex of long-chain fatty acyl-CoA and ACBP is transported either into mitochondria for β-oxidation (70-80%) or the fatty acyl-CoAs are esterified (10-30%) mainly to form triacylglycerols (TG) (Fig. (**1**)).

Assuming that one potential mechanism of myocardial lipotoxicity may be the result of a mismatch between lipid oxidation and lipid uptake, it must be acknowledged that changes in mitochondrial LCFA oxidation may therefore affect triacyglycerol (TG) stores and lipid deposition. A possible role for FAT/CD36 in contribution to the regulation of mitochondrial LCFA utilization has been recently proposed as we [47,48] have found that FAT/CD36 is present in the mitochondrial membrane in skeletal muscles. Furthermore, during increased energy demands of the skeletal muscle (electrically-stimulated contraction or exercise) FAT/CD36 was translocated, presumably from an endosomal pool to the mitochondria [47,49]. A functional role for FAT/CD36 was strongly suggested, because sulfo-*N*-succinimidyl-oleate (SSO), a known covalent inhibitor of FAT/CD36 [28], almost fully inhibited mitochondrial fatty acid oxidation, either when muscle was at rest or had been contracting [47-49].

## REGULATION OF CARDIAC LCFA UPTAKE

*In vivo*, several factors have been implicated in the regulation of the blood-borne LCFA entry into cardiac myocytes including (1) availability of exogenous substrates (substrate milieu); (2) hormone milieu (particularly insulin); (3) cardiac energy demands (workload/heart rate); and (4) an adequate oxygen supply.

### Regulation by the Hormonal Milieu

It has been shown that acute and chronic exposure to insulin favors LCFA uptake and subsequent esterification in cardiac myocytes [15]. Acute insulin exposure (30 min) stimulated the rate of LCFA transport into cardiac myocytes by inducing the translocation of only FAT/CD36, but not FABPpm nor FATP1, from an intracellular membrane compartment to the plasma membrane (Fig. (**2**)). This suggests that FABPpm and FATP-1 play minor or permissive roles in LCFA trans-sarcolemmal transport into cardiac myocytes when PI3 kinase is activated by insulin [12,13,15]. Chronic exposure of quiescent cardiac myocytes to insulin (2h) not only induced FAT/CD36 translocation, but also increased FAT/CD36 expression at the protein level [12] and Fig. (**3**). An insulin-induced increase in plasmalemmal FAT/CD36 stimulated the rate of palmitate transport into cardiac myocytes, which paralleled the increase in the content of myocardial lipid pools (mainly PL and TG stores) [15].

It has been suggested that other hormones are likely also involved in the regulation of intracellular lipid homeostasis. One of the most potent candidates includes leptin [50]. In isolated working rat hearts leptin increased the rate of palmitate oxidation [44] but this effect appeared to occur independently of the well-known leptin-induced activation of the AMPK-ACC-malonyl-CoA (AMP kinase and acetyl-CoA carboxylase) axis [51]. Interestingly, in our laboratory, we observed that leptin-induced stimulation of LCFA oxidation in cardiac myocytes is critically dependent on the leptin-induced increase in LCFA transport *via* the translocation of FAT/CD36 [Bonen *et al.* unpublished observations].

### Regulation by Contraction and AMPK Activation

It is well known that myocardial energy metabolism and LCFA utilization are substantially increased [16] when the workload of the heart is increased or when quiescent cardiac myocytes are electrically stimulated to contract. Indeed, LCFA taken up into contracting cardiac myocytes were efficiently channeled into mitochondrial β-oxidation, while esterification into cellular lipid pools remained unaltered [16]. Recent studies have revealed that during electrically-induced contraction of cardiac myocytes, the translocation of FAT/CD36 from intracellular pool(s) to the plasma membranes is a major mechanism responsible for the increase in LCFA uptake rate [16]. It was also demonstrated that the contraction-induced increase in FAT/CD36-mediated LCFA uptake was completely inhibited by SSO, confirming the involvement of FAT/CD36 in the underlying mechanism of LCFA transmembrane transport [16,28].

Increased cardiac workload is known to stimulate components of several intracellular signaling pathways [52,53]. Thus, the contraction-induced translocation of FAT/CD36, and subsequent increase in LCFA transport, might be linked to the activation of AMP kinase, protein kinase A (PKA), members of the protein kinase C (PKC) family, or mitogen-activated protein kinase (MAPK). Our work has recently demonstrated that electrical stimulation of quiescent cardiac myocytes activates AMPK, which results in the translocation of FAT/CD36 to the plasma membrane and a concurrent increase in the rate of LCFA transport [16]. Similarly, pharmacological AMPK activation in perfused heart, by contraction-mimetic compounds (5-aminoimidazole-4-carboxamide-1-ß-D-ribofuranoside-AICAR) induced the translocation of both FAT/CD36 and FABPpm to the plasma membrane, and as a result, LCFA transport was increased [13,54]. Both transporters appeared to contribute to the AICAR-mediated increase in LCFA uptake (Fig. (**2**)). However, this notion is complicated by the observation that the FAT/CD36 inhibitor SSO completely blocked the AICAR-induced LCFA uptake, suggesting that the FABPpm component in AICAR-induced LCFA uptake is dependent on FAT/CD36 [54]. Furthermore, we have recent evidence suggesting a direct interaction between FAT/CD36 and FABPpm at the myocardial sarcolemma [Chabowski A and Bonen A unpublished observations]. These observations seem to imply that when cellular energy demands are challenged (AMP/ATP ratio is increased), the LCFA transport system is activated by means of translocation of both transporters (FAT/CD36 and FABPpm) to the sarcolemma, which results in greater LCFA influx, which allows more LCFAs to be delivered to mitochondria. Thus, an increased rate of LCFA transport serves to provide the necessary substrate supply to support an increase in mitochondrial β-oxidation in the heart.

### Alternate Signaling Pathways

While insulin signaling, *via* the activation of PI3-kinase [15] and AMPK activation by contraction [16] provide signals for increasing LCFA transport, it is now also possible to exclude several signaling pathways in stimulating LCFA transport. Specifically, we have recently shown that cyclic AMP dependent protein kinase A (PKA) is not involved in regulating LCFA uptake by FAT/CD36 [55] because, in quiescent cardiac myocytes, pharmacologically-increased levels of intramyocardial cAMP failed to stimulate the translocation of LCFA transporters. It also appears that the MAPK signaling cascade is not involved in the acute regulation of LCFA transporter translocation and subsequent LCFA myocardial uptake [56]. However, separate stimulation of PKC by the phorbol ester 12-myristate 13-acetate (PMA) resulted in an increased rate of LCFA uptake in isolated cardiac myocytes; this effect was blunted by SSO, indicating that certain PKC isoforms enhance LCFA uptake in a CD 36-dependent manner [55]. Clearly, the signaling pathways involved in recruiting LCFA transporters to the cell surface are only now beginning to be explored. It will be important to deduce which signaling pathways are important and whether there are different signaling pathways for different LCFA transporters.

### Adequate Oxygen Supply and LCFA Myocardial Metabolism

Myocardial metabolism of LCFA is highly dependent on adequate oxygen supply. In the ischemic myocardium, LCFA are diverted from β-oxidation into deposition as neutral intracellular lipids within the tissue [57,58]. This myocardial lipid accumulation during hypoxia could be at least partially due to the mismatch between a reduced rate of β-oxidation and the increased rate of LCFA transport into cardiac myocytes, the latter occurring as a result of the low oxygen-induced translocation of FAT/CD36 and FABPpm to the plasma membrane [59] and Fig. (**4**). Lipid accumulation in the ischemic heart is likely also related to the switch in energy substrate preference from the predominant oxidation of LCFA occurring in healthy heart to the increased utilization of glucose during heart failure [2,60,61]. This is the case when cardiac myocytes are exposed to a lower oxygen supply over a prolonged period of time. It has been suggested that there is a direct involvement of LCFA transporters in the transition away from LCFA metabolism in long-term heart failure [62]. For example, in infarcted rat hearts, an animal model of heart failure, there are parallel reductions in the total content of LCFA transporters (FAT/CD36, FABPpm, FATP-1 and FATP-6) and palmitate oxidation, along with a concomitantly reduced cardiac ejection fraction [62]. A marked decrease in myocardial LCFA oxidation was also observed in FAT/CD36 null mice, suggesting that there was a significant energy impairment (decreased ATP production) when hearts were challenged with low-flow ischemia [18]. However, a recent study strongly points to the fact that FAT/CD36 deficient hearts are not energetically or functionally impaired, most likely due to compensatory increase in glucose oxidation. Importantly, FAT/CD36 deficient hearts, after low-flow ischemia, recovered to the same extent as wild type hearts [19].

### Regulation of Protein-Mediated LCFA Transport by Substrate Milieu

Cardiovascular abnormalities remain the leading cause of mortality in individuals with obesity and obesity related type 2 diabetes. It is widely accepted that hyper-and/or hypo- insulinemia, hyperglycemia, and increased plasma LCFA and/or lipoprotein concentrations are associated with obesity and type 2 diabetes, and may negatively influence myocardial performance [26,63,64].

Blood plasma concentrations of metabolic substrates have a significant impact on myocardial metabolism [65], because it is known that selected substrates can regulate the expression of specific metabolic genes [66-69]. LCFA`s are known ligands for the peroxisome proliferator-activated receptors (PPARs), which regulate genes involved in fatty acid metabolism [70,71]. Consistent with their roles in LCFA transport, LCFA transporters appear to be upregulated when the LCFA milieu is increased by a high fat diet or by fasting [72]. For example, FAT/CD36 mRNA was upregulated in neonatal cardiac myocytes after a 48 h exposure to high concentrations (0.25 mM) of palmitate [73]. Moreover, recent findings indicate that in quiescent cardiac myocytes palmitate exposure (2h) (0.1-1mM) also resulted in an increased expression of FAT/CD36 protein, which appeared to be due to posttranscriptional events [Chabowski A *et al.* unpublished data].

Data obtained from rodent studies suggest that in health, the balance between the myocardial utilization of the main metabolic substrates is finely tuned (i.e. LCFA ~70% and glucose ~30%). This, however is disturbed in cardiac disease, so that a marked preference for largely a single substrate (glucose or LCFA) arises, which can result in cardiac malfunctioning [26]. Specifically, altered myocardial substrate metabolism, namely reduced rates of glucose use (glycolysis and glucose oxidation) with a concomitant increase in rates of LCFA oxidation are primarily responsible for diabetic cardiomyopathies [26,74-77]. Furthermore, since it was shown that, disturbance in cardiac myocyte lipid utilization preceded the development of cardiomyopathy, it appears that despite increases in LCFA oxidation, lipid accumulation predominates and in the myocardium plays a central role in the etiology of diabetic cardiomyopathy [78,79]. It has become evident that when the LCFAs supply exceeds the heart’s capacity for their utilization, accumulation of intracellular lipids leads to arrhythmias, cardiac myocyte apoptosis and congestive heart failure. For instance, Schaffer’s group [80] has demonstrated that the mismatch in LCFA uptake and oxidation (uptake >> metabolism) contributed to lipotoxicity in the heart, resulting in cardiac triacylglycerol accumulation, and left ventricular dysfunction and premature death. Thus, therapies aimed at removing the excess of intracellular LCFA from the cytosol should be beneficial, since high concentrations of myocardial LCFA are detrimental to proper cardiac functioning [81,82].

## CARDIAC FATTY ACID TRANSPORTERS IN DIABETES, INSULIN RESISTANCE AND OBESITY

The pathogenesis of type 2 diabetes and insulin resistance have both genetic and environmental (diet, physical activity) contributions. Animal genetic models of the type2 diabetes almost exclusively involve leptin disruption or its receptor dysfunction [for review: 63]. Models of insulin resistance (e.g. high-fat or high-carbohydrate diets) result only in mild hyperglycemia [83,84]. Nevertheless in these models cardiac LCFA oxidation is altered. For example, in type 2 diabetic animal models, such as db/db or insulin resistant ob/ob mice the data consistently point to an increased FA oxidation in heart [75-77]. This is also found in and Zucker diabetic fatty rats [85]. It has also been noted that there is an increased intracellular TG content in the hearts of ob/ob mice and ZDF rats [86]. Collectively, these studies strongly imply that there is an imbalance between FA oxidation and fatty acid uptake in the heart of diabetic animal models. This has now been shown in several studies. In skeletal muscle and hearts of type 2 diabetic rats and mice, fatty acid transport rates were increased as a consequence of an increased plasmalemmal FAT/CD36 content [74, 92]. That fatty acid transporters were the key factor in upregulating LCFA oxidation in hearts of type 2 diabetic *db/db* mice was recently shown. In these mice, it was the increase in plasmalemmal FAT/CD6 and FABPpm that accounted for the increased rates of LCFA esterification and oxidation, since no differences in any other parameters in LCFA metabolism were apparent in this model of type 2 diabetes Fig. (**6**) and [92].

In a model of obesity and insulin resistance, namely obese Zucker *fa*/*fa* rats, TGs and ceramides accumulate in the heart. This is not only the consequence of an increase in plasma lipids, but is also attributable to the shift in cardiac LCFA metabolism. In this model of obesity LCFA uptake into the myocyte is increased, LCFA oxidation is not altered while LCFA esterification is markedly increased [78]. Concurrently, glucose and lactate oxidation are inhibited [78]. This type of substrate switch, such as occurs in obese Zucker rats, was shown to be associated with the development of contractile dysfunction [78]. Chronically increased FA utilization is thought to contribute to ventricular dysfunction by increasing mitochondrial oxygen consumption, generation of reactive species due to increased flux through oxidative pathways, or *via* the toxic effects of accumulated lipid species [78-80]. A role for fatty acid transporters in obesity-related myocardial lipid accumulation is evident in obese Zucker rats. For example, in insulin resistant, obese Zucker rats we found that myocardial FAT/CD36 protein expression was not altered, but instead it was permanently relocated to the cardiomyocyte plasma membrane, leading to an increase in LCFA transport and subsequent channeling of LCFA into triacylglycerols Fig. (**5**) and [90]. Thus, cardiac myocyte lipotoxicity likely is the result of LCFA uptake being in excess of its disposal rate (utilization) through oxidation, thereby leading to excessive lipid accumulation [90].

Another animal model that exhibit myocyte lipid accumulation and cardiac dysfunction has recently been described [87]. In this model mice with cardiac-specific over-expression of PPARα present enhanced expression of FA metabolizing genes with a concomitant increase in FA oxidation. These alterations resemble those in animal models of obesity and diabetes. Furthermore, cardiomyopathy in this model can be exacerbated by increasing the circulating non-esterified fatty acids (high fat diet) [88] or by diminishing FAT/CD36 (this increases circulating fatty acids), when MHC-PPARα mice were crossed with mice deficient for CD36 [89]. 

We have also examined the effects of two levels of STZ-induced diabetes (moderate and severe) on LCFA transport and LCFA transporter (FAT/CD36 and FABPpm) expression, at the mRNA and protein level, as well as their plasmalemmal localization [91]. These studies have shown that with STZ-induced diabetes LCFA transport across the plasma membrane is increased in heart in relation to the severity of insulin deficiency (severe diabetes > mild diabetes). These increases in LCFA transport in heart were associated with concomitant increases in the expression of FAT/CD36 and FABPpm proteins, which resulted in the increased presence of FAT/CD36 (moderate and severe diabetes) and FABPpm (severe diabetes only) at the sarcolemma [91]. 

Collectively, a number of studies have clearly shown the importance of LCFA transporters in regulating LCFA uptake and metabolism in the heart, in models of type 1 and type 2 diabetes, and in obesity. It is however difficult to determine whether the increases in sarcolemmal LCFA transporters, and the subsequent enhancement in LCFA transport, are related to hyperglycemia or hypoinsulinemia, or to other systemic factors that are disturbed, since *in vivo* a great many factors contribute to the regulation of lipid metabolism. Nevertheless, Chen *et al*. [93] have recently implicated hyperglycemia as a key factor, since vanadate-induced normalization of circulating glucose in STZ-induced diabetic rats, resulted in a significant reduction in FAT/CD36 mRNA abundance [93]. Similarly, Griffin *et al*. have shown increased translation of macrophage CD36 transcript under high glucose conditions [94]. A better understanding of how the independent or combined availability of substrates (LCFA, glucose, lactate) regulate the expression and subcellular localization of LCFA transporters in the heart remains to be established.

## CARDIAC FATTY ACID TRANSPORTERS AND CARDIOVASCULAR DISEASES IN HUMANS

Based on data from rodent models, human gene CD36 mutations have been implicated to play a causal role in impairment of insulin action. A relatively high frequency polymorphism in human FAT/CD36 occurs in Asian and African populations, and these have been associated with reductions in LCFA myocardial uptake and associated cardiac abnormalities [95,96]. For example, in CD36-deficient patients a high correlation was found between the reduction in the uptake of the iodinated LCFA analogue BMIPP into the heart and the increase in glucose oxidation [97-100]. Paradoxically, it has also been shown that human CD36 deficiency co-exists with insulin resistance [97]. However, these studies in humans are limited and confusing, as essentially FAT/CD36 overexpression in rodent models has commonly been linked to intracellular lipid accumulation, and therefore, one would expect that an FAT/CD36 deficiency might contribute to limiting intracellular lipid storage. One possibility is that the observed insulin resistance in these patients is associated with an increased hepatic uptake of LCFAs, involving fatty acid transporters other than FAT/CD36 [101,102].

## SUMMARY

LCFA transporters (FAT/CD36 and FABPpm) play a pivotal role in the transport of LCFAs across the plasma membrane and thereby these proteins contribute to the regulation of energy provision in cardiac muscle cells. In addition, it is likely that cardiac myocyte lipotoxicity is associated with either the increased expression of LCFA transporters and/or with their permanent relocation to the plasma membrane. This is the case when LCFA oxidation rates remain constant or are enhanced but not in proportion to the increased rates of LCFA transport (i.e. transport > oxidation). Thus, additional studies are needed to better understand the regulation of LCFA uptake into the myocardium and the subsequent partitioning between oxidative and nonoxidative metabolic pathways.

## Figures and Tables

**Fig. (1):**
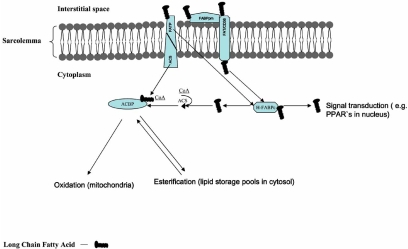
Schematic view of protein-mediated, long-chain fatty acid uptake and metabolism in cardiac myocytes. Abbreviations: FAT/CD36 - fatty acid translocase; FABPpm - plasmalemmal fatty acid-binding protein; FABPc - cytoplasmic fatty acid-binding protein; FATP - fatty acid transport protein; ACBP - acyl-CoA binding protein; ACS - acyl-CoA synthetase. For clarity the scheme shows FAT/CD36 and FATP1 to function independently, this however is not known for certain.

**Fig. (2):**
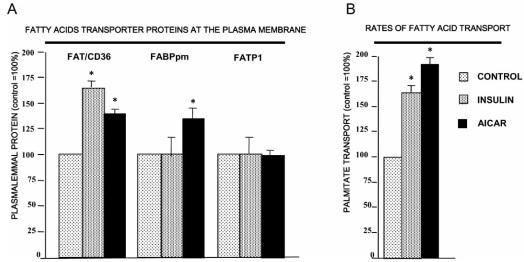
Acute (30 min) effects of insulin (10 nM) and AMP kinase activation by AICAR (2 mM) on the content of plasmalemmal LCFA transporters (A) (FAT/CD36, FABPpm and FATP-1) and on the rate of palmitate transport (B) in rat cardiac myocytes (FAT/CD36, FABPpm and FATP-1) (Redrawn from data published by Chabowski *et al*. [13]).

**Fig. (3):**
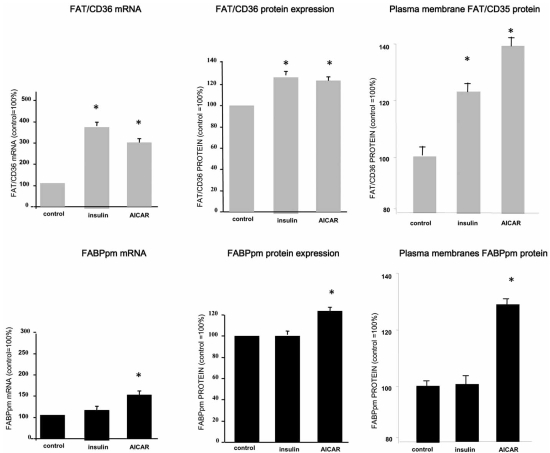
Chronic (2h) effects of insulin (10 nM) and AMP kinase activation by AICAR (2 mM) on the transcription (mRNA), total protein and plasmalemmal expression of FAT/CD36 and FABPpm in isolated rat cardiac myocytes. (Redrawn from data published by Chabowski *et al*. [12,54]).

**Fig. (4):**
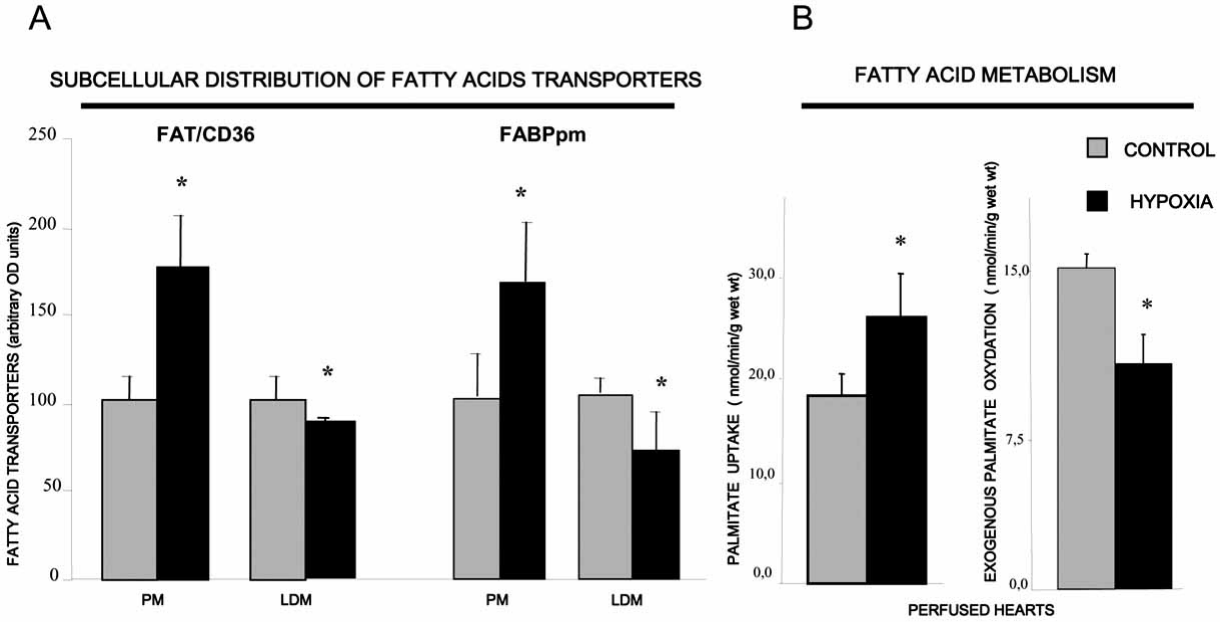
Changes in fatty acid transporters (FAT/CD36 and FABPpm) plasmalemmal content and FA transport (A) and oxidation induced by acute hypoxia (15 min) (B). PM- plasma membranes, LDM-low density microsomes. (Redrawn from data published by Chabowski *et al..* [59]).

**Fig. (5):**
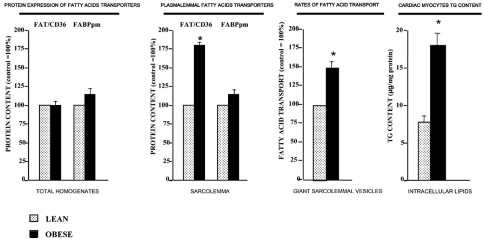
Changes in fatty acid transport, and FAT/CD36 and FABPpm protein expression and plasmalemmal content in hearts of obese Zucker rats. For each parameter the control has been set to 100%. Note that fatty acid transporter expression is not altered but that fatty acid transport is up regulated in concert with an increase in plasmalemmal FAT/CD36, indicating that FAT/CD36 is permanently relocated to the plasma membrane (obese Zucker rat). (Redrawn from data published by Coort *et al.* [90] and Luiken *et al.* [103]).

**Fig. (6):**
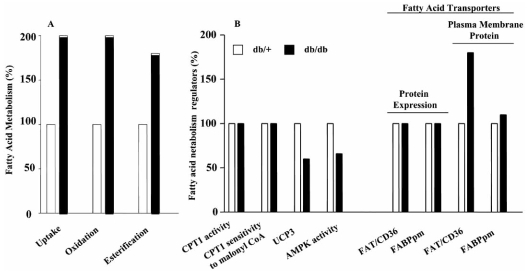
Cardiac fatty acid uptake and metabolism in db/+ and db/db mice (A) and selected factors known to influence fatty acid oxidation (B). Note that the only factor accounting for the increased rate of fatty acid oxidation is the content of plasma membrane FAT/CD36 (data redrawn from Carley *et al.* [92]).
